# *Ageratina adenophora* induces mice hepatotoxicity via ROS-NLRP3-mediated pyroptosis

**DOI:** 10.1038/s41598-018-34492-7

**Published:** 2018-10-30

**Authors:** Wei Sun, Chaorong Zeng, Shanshan Liu, Jie Fu, Liwen Hu, Zhen Shi, Dong Yue, Zhihua Ren, Zhijun Zhong, Zhicai Zuo, Suizhong Cao, Guangneng Peng, Junliang Deng, Yanchun Hu

**Affiliations:** 1 Key Laboratory of Animal Diseases and Environmental Hazards of Sichuan Province, College of Veterinary Medicine, Sichuan Agricultural University, Wenjiang District, Chengdu City, Sichuan 611130 China; 2 Tongren Polytechnic College, Bijiang District, Tongren City, Guizhou 554300 China; 3 Sichuan China 81 Rehabilitation Center, Sichuan Provincial Rehabilitation Hospital, Chengdu, Sichuan 611135 China

## Abstract

Increasing evidences have demonstrated that *Ageratina adenophora* (*A. adenophora*) can cause hepatotoxicity of animals. Liver is an important site in immune regulation and inflammatory responses. However, the information about hepatotoxicity induced by *A. adenophora* in relation to inflammation is still finite. To investigate the underlying mechanism, we conducted animal experiments with different dosage of *A. adenophora*. Mice were randomly divided into 4 groups and administrated with 0%, 10%, 20% and 30% levels of *A. adenophora* pallet diet in control, group A, B and C, respectively. The results showed that *A. adenophora* caused hepatotoxicity as revealed by increasing alkaline phosphatase, alanine aminotransferase, aspartate aminotransferase. Then, the reactive oxygen species (ROS) levels were shown to be elicited by *A. adenophora* through flow cytometry assay in a dose-dependent manner. Furthermore, pyroptosis was activated by *A. adenophora*, which was characterized by increasing protein and mRNA levels of caspase-1, gasdermin D and interleukin-1β. Notably, ROS down-stream factors, including nod-like receptor inflammasome protein 3 and nuclear factor-κB, were also activated by *A. adenophora*. These data demonstrated that *A. adenophora* caused liver inflammatory injury and induced hepatocyte pyroptosis by activating NLRP3 inflammasome, which was triggered by elevating ROS production levels. This research might provide new insights into the mechanism of hepatotoxicity induced by *A. adenophora*.

## Introduction

*Ageratina adenophora* (*A. adenophora*, also called as *Eupatorium adenophorum*), originating from Mexico, is a perennial semi-shrubby herbaceous plant, which has successfully invaded into many countries^1^. In China, *A. adenophora* first invaded in Menghai county, Yunan province from Burma and Vietanam in 1940, and it dispersed to northwards and eastward at an annual average speed of 20 km^2^. It is estimated that the area of invasion in China is currently more than 30 million hectares^3^. And the general economic losses caused by *A. adenophora* to husbandry and grassland ecosystem have been estimated at RMB 3.62 billion per year^3,4^. Due to the damaged caused by *A. adenophora*, it has become the most destructive invasive species in China, especially in the southwestern regions^5^. *A. adenophora* is troublesome specie in the invasive areas, which can encroach on grass and cause livestock poisonings, including acute asthma, diarrhea, depilation, even death^6^. Accumulating researchers have demonstrated that hepatotoxicity induced by *A. adenophora* in several species of animals^7–10^. Even so, the scientific basis for toxicological effects caused by *A. adenophora* is poor-elucidated, and the underlying molecular mechanism is still limited.

Liver plays a primary role in the detoxification of ingested toxin, as well as a major site for regulation of immune due to its unique function and anatomical location^11^. Here, for the first time, we evaluated the hepatotoxicity induced by *A. adenophora* in relation to inflammation. Inflammasome has a pivotal role in initiating of immune responses by providing platforms for the activation of inflammatory-associated caspase proteases^12^.

Reactive oxygen species (ROS), as the byproducts of normal cellular metabolism, regulates the signaling pathways in response to the changes of the intracellular and extracellular environments^13^. However, overproduction of ROS may behave as poisonous and toxic products which induces dysfunction of cell and tissue^14^. The increased ROS could promote the release of inflammatory-related signaling factors, including nod-like receptor inflammasome (NLRs) and nuclear factor-κB (NF-κB)^15^. The activated NLRP promotes the maturation of pro-caspase-1, resulting in a novel cell death named pyroptosis, which characterizes by pore formation of the plasma membrane and cell swelling^16,17^.

Pyroptosis, a new programmed cell death, has an inherent of pro-inflammatory character, which can be triggered by a variety of inflammasome complexes^18–20^. Previous studies have demonstrated that pyroptosis is emerging as a ubiquitous immune effector in a variety of cells^18,21^. It can be triggered by various infections and non-infections stimulates^22^. In the process, cells recognize foreigner signals and secrete pro-inflammatory cytokines as well as release intracellular contents^23^. Basing on our previous study and the toxicological effects of *A. adenophora*, we hypothesized that pyroptosis induced by ROS-mediated NLRP3 activation pathway might play a fundamental role in the process of hepatotoxicity induced by *A. adenophora* in mice hepatocytes.

## Results

### Gross lesions

**Figure 1:**
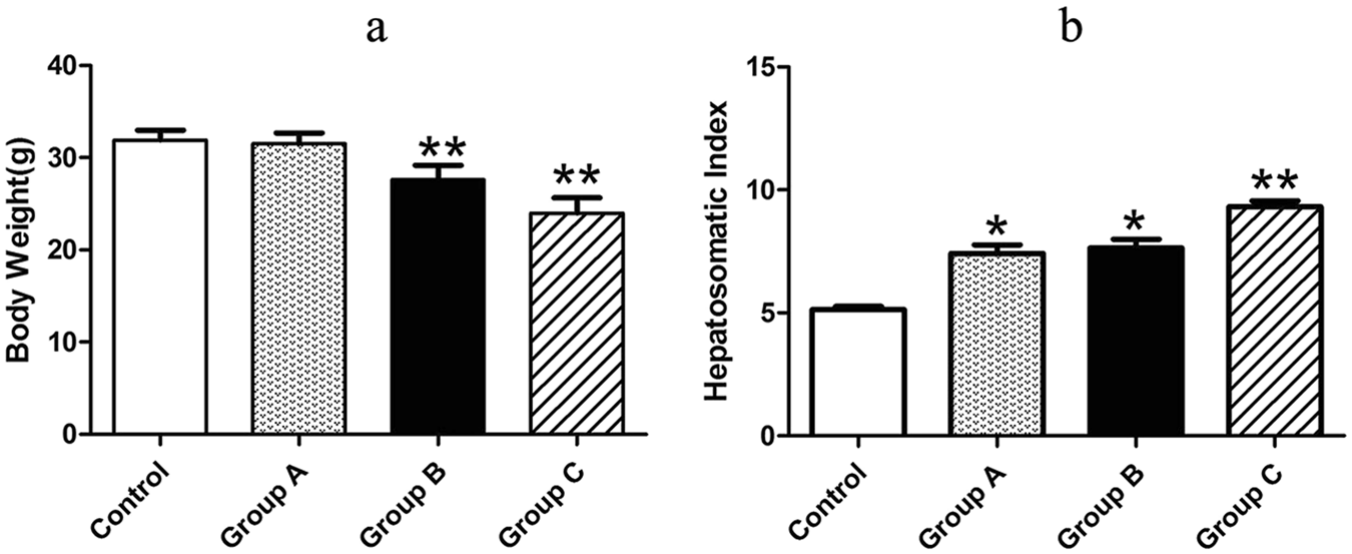
Changes of BW and HIS after *A. adenophoraA. adenophora* administration. (**a**) Changes of mice BW in control and *A. adenophora* exposure group. The BW in *A. adenophora*-treated groups was decreased in dose-dependent manner compared with control group. Data are represented as mean ± SD, n = 6; **p < 0.01. (**b**) The changes of HIS in different groups. The data are represented as mean ± SD, n = 5. *p < 0.05 and **p < 0.01 vs. the control group.

The mice were carefully observed for their psychosis and activities in the whole experiment period. The mice in *A. adenophora* administration groups appeared different degree of clinical signs, such as drowsiness, ataxia, roughened hair and other toxic symptoms, but these phenomena not appeared in the control group. The body weight (BW) was decreased in a dose-dependent manner in comparison with the control group (Fig. 1a).

The hepatosomatic index (HIS) was carried out as previous described to evaluate the morphological change of the livers induced by *A. adenophora* exposure^24^. The results showed that HSI in the *A. adenophora* administrated groups were all remarkably higher than that in the control group (Fig. 1b), indicating obvious hepatomegaly caused by *A. adenophora*.

### Biochemical indices assay

**Figure 2:**
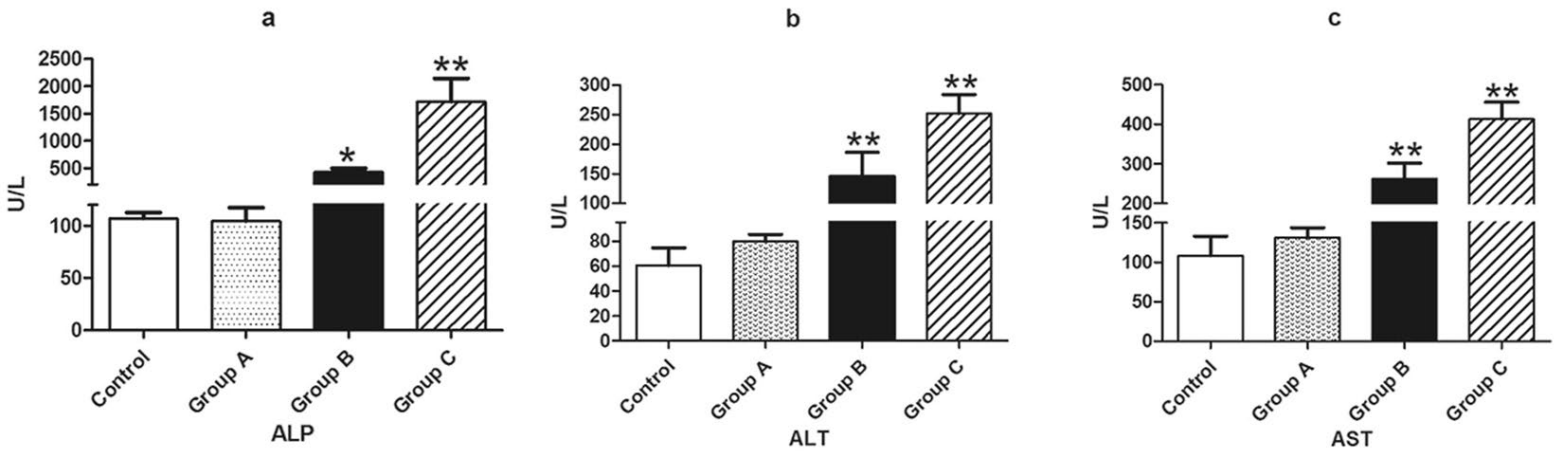
Biochemical indices changes in per group after *A. adenophora* administration. ALP, ALT and AST were increased in group B and C dose-dependently. Significant different from control group. The histograms are representive of 3 separated experiments. The data are represented as mean ± SD of three independent experiments. **p < 0.01 in compared with the control group.

The biochemical examination was performed to investigate the change of cellular and metabolic substances^8,25^. The detection and analysis of enzymatic spectrum in serum have a great significant in evaluating the degree of organ damage caused by toxic effects. In the present study, alkaline phosphatase (ALP), alanine aminotransferase (ALT), aspartate aminotransferase (AST) were used to evaluate the toxicity of *A. adenophora* on mice liver (Fig. 2a–c). Compared with control group, ALP, ALT and AST in group B and group C were significantly increased. These biochemical parameters above mentioned were elevated by *A. adenophora* in dose-dependent manner (Fig. 2).

### *A. adenophora* treatment increases ROS levels in mouse hepatocytes

**Figure 3:**
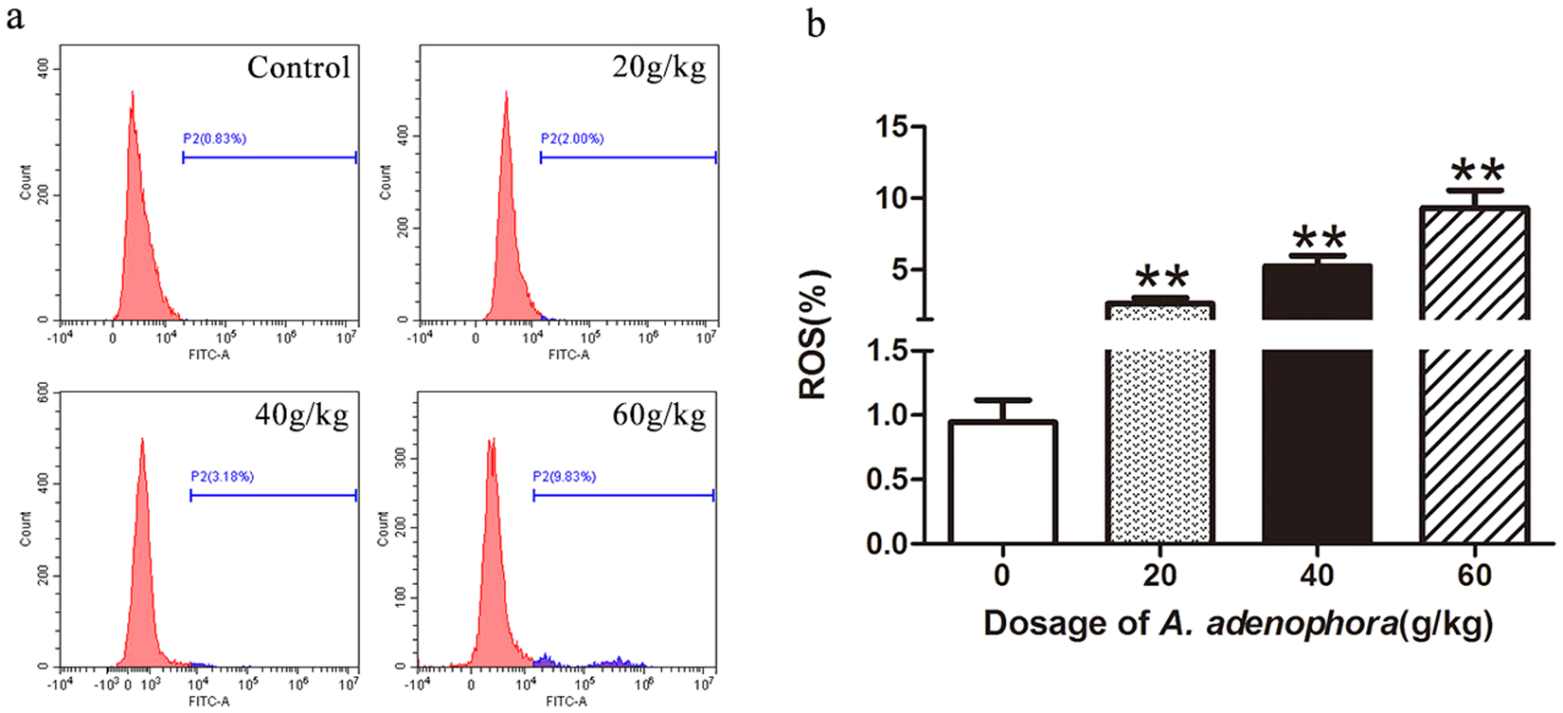
Effects of *A. adenophora* on ROS production in hepatocyte. (**a**) Mice were treated with different dosages of *A. adenophora* for 6 weeks. Subsequently, the hepatpcyte ROS levels were analyzed by flow cytometry in the FITC channel. (**b**) Quantitative measured of ROS positive cell in six independent experiments, data are represented as mean ± SD. **p < 0.01 vs. the control group.

Overproduction of free radicals or oxidant species in cell and tissue, behave as toxic and deleterious substances, which will lead to cellular and tissular dysfunction. Therefore, we measured the intracellular ROS production levels in the control and *A. adenophora*-treated murine hepatpcytes through flow cytometry (Fig. 3a). In comparing with control group, significant increasing levels of ROS were observed in all *A. adenophora* administration group (Fig. 3b). These data indicated that *A. adenophora* posed hepatotoxicity by elevating ROS level.

### Detection of pyroptosis in hepatocyte

**Figure 4:**
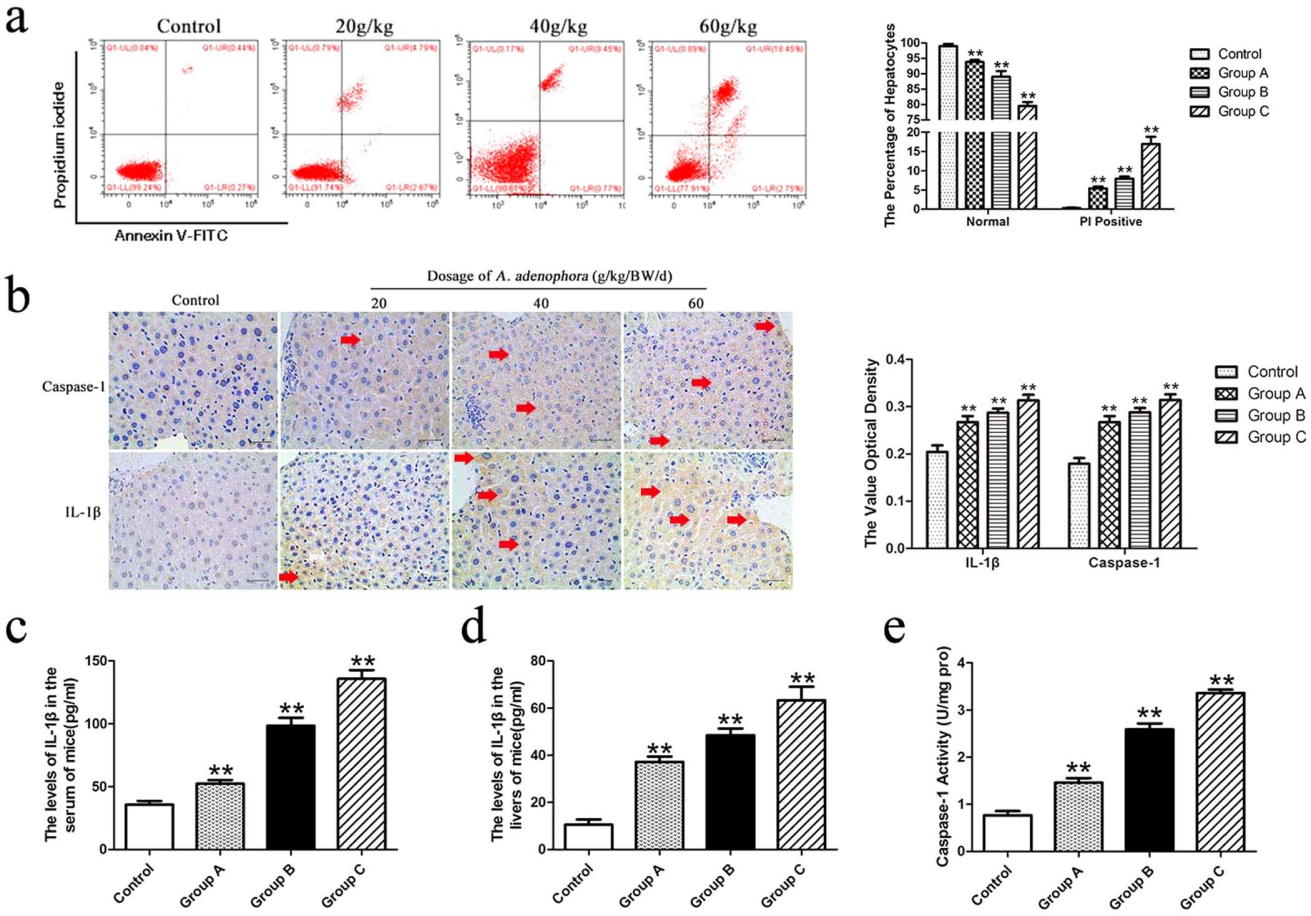
*A. adenophora* induced in pyroptosis hepatocyte. (**a**) Scattergram of pyroptosis of hepatocytes. The hepatocytes were analyzed for pyroptosis through flow cytometry basing on Annexin V-FITC/PI staining. *A. adenophora* markedly increased PI positive cell in dose-dependent manner. (**b**) Caspase-1 and IL-1β expression were detected by immunohistochemistry method. The optical density values were counted by three random microscope. Magnification, 400×. Scale bar = 40 μm. The distribution of caspase-1 and IL-1β (yellow stain regions were indicated by red arrows) were increased with the increasing level of *A. adenophora*. ELISA was used to measured the levels of IL-1β in serum and liver homogenate (**c**,**d**). Caspase-1 activity was measuredindifferentgroups (**e**). The data were analyzed using Student’s t test and were considered signifcant as follows: **p < 0.01. All data are represented as mean ± SD.

Propidium idodide (PI) is a kind of nuclei acid dye, which can not to penetrate the whole cell membrane, but the damaged cell membrane of pyroptosis^26^. The hepatocytes were assessed through flow cytometry using Annexin V-FITC/PI staining (Fig. 4a). According to the flow cytometry analysis, the normal hepatocytes percentages in *A. adenophora*-treated groups were significantly decreased by comparing with control group. Wheresas the percentage of PI positive pyroptosis hepatocytes in group A, B and C were significantly increased with the increasing dose of *A. adenophora* administration (Fig. 4a). Generally, pyroptosis is activated by caspase-1 dependent canonical pathway. To identify whether caspase-1 dependent pathway was activated by *A. adenophora*, two key pyroptosis factors were assayed by immunohistochemistry method. As shown in Fig. 3b, the optical density value of caspae-1 and IL-β in liver were enhanced in a dose-dependent manner. By comparing with the control group, the production levels of IL-β in both serum and liver homogenate were elevated in *A. adenophora*-treated group (Fig. 4c,d). Moreover, caspase-1 activity was also augmented by *A. adenophora* (Fig. 4e).

### Expression of inflammation and pyroptosis related genes and protein in murine liver

**Figure 5:**
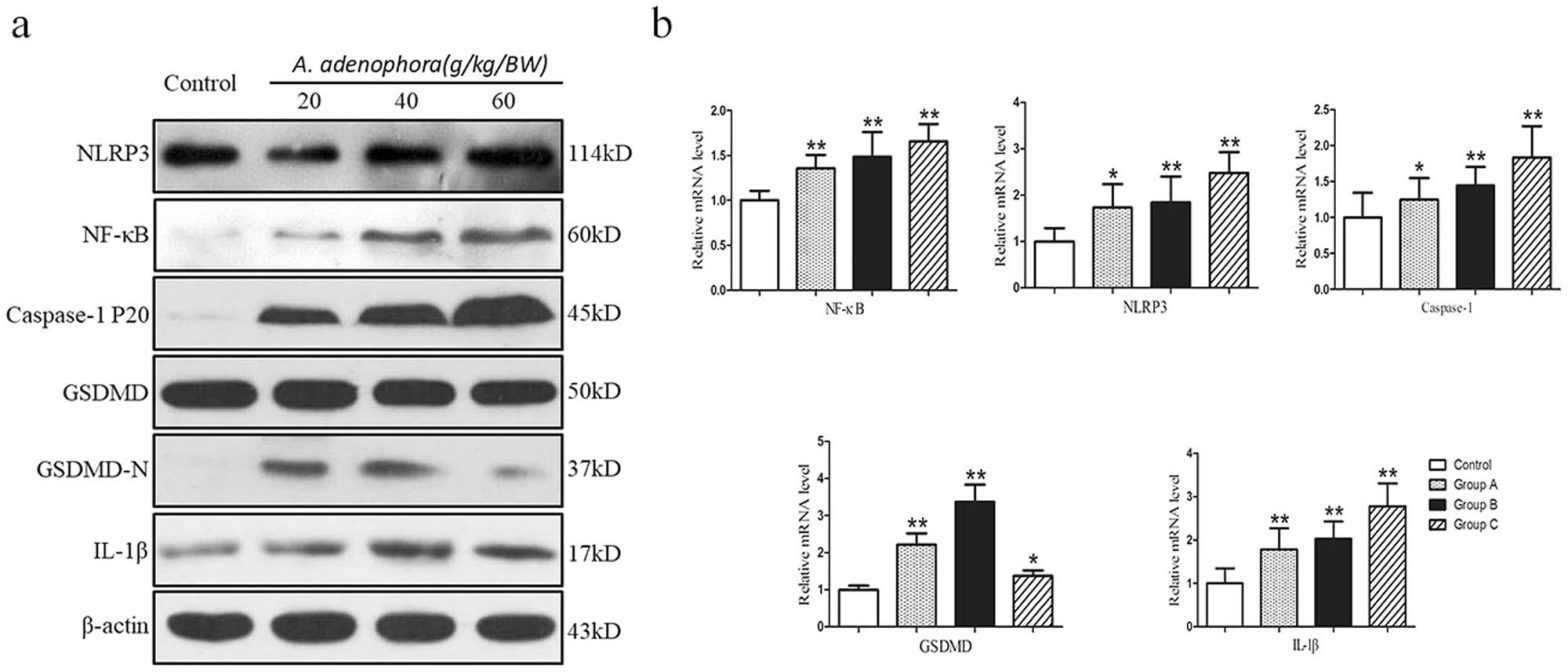
Detection of protein and genes levels of hepatotoxicity associated in liver. (**a**) Western blot was performed to detect the protein expression levels of NLRP3, NF-κB, Caspase-1, GSDMD and IL-1β in liver, and β-actin was serviced as conrtol. Blots was exposured 1–3 mins and cropped to show the relevant parts only and full images are provided in Supplementary Fig. S1. (**b**) The mRNA expression levels of NLRP3, NF-κB, Caspase-1, GSDMD and IL-1β were measured by the 2^−ΔΔCt^ method with relative quantification. *p < 0.05, **p < 0.01. The data are showed as mean ± SD.

To further detect the influence of *A. adenophora* on pyroptosis, the protein and mRNA level of caspase-1 and IL-1β were measured by western blot and qRT-PCR, respectively (Fig. 5). These results showed that caspase-1 protein was activated with the increasing levels of *A. adenophora*. (Fig. 5a), and the mRNA level of caspase-1 was significant up-regulated by *A. adenophora* (Fig. 5b). In addition, IL-1β in both protein and transcriptional level were increased by *A. adenophora* (Fig. 5). Gasdermin D (GSDMD), the downstream factor of caspase-1, is key executioner in pyroptosis pathway. The formation N-terminal of GSDMD (GSDMD-N), a cleavage body of GSDMD, promotes membrane rupture that causes IL-1β release. Western blot results showed that GSDMD-N occurred in *A. adenophora*-treated groups, but not in the control group (Fig. 5a). And the mRNA levels were all elevated by *A. adenophora* than that in the control group (Fig. 5b). Those data demonstrated that pyroptosis was induced by *A. adenophora* through caspase-1 dependent pathway.

NLRP3 could be triggered by a variety of activators. Studies showed that ROS was generated by NLRP3 activator which drove inflammasome activation^15^. In this study, we found that *A. adenophora* promoted the increasing of ROS (Fig. 3). To confirm whether NLRP3 could be activated by *A. adenophora*, the mRNA and protein expression were measured by qRT-PCR and western blot, respectively. As shown in Fig. 5, NRLP3 was activated by different dose of *A. adenophora*. And another ROS down-stream factor, NF-κB, as well as the activator of caspase-1, was also elevated in protein and mRNA levels.

## Discussion

*A. adenophora* is a noxious alien invasive species that spread all over the world^27^. Previous studies showed that the ingestion of *A. adenophora* as a feed additive induced hepatotoxicity in mice^28^ and rat^7^, as well as chronic pulmonary disease in horses^29^. In our previous studies, apoptosis pathway was triggered by *A. adenophora* in goat and mice^10,30,31^. In this study, IL-1β, a pro-inflammatory cytokine, was induced by *A. adenophora* (Fig. 4c,d), suggesting that inflammation was presented in the liver, which was in accordance with previous reports in the literatures^25,32^. However, apoptosis is known as a non-lytic form of cell death, which is described as an active programmed process of cell division that avoids triggering inflammation^33,34^. This discrepancy might be caused by of a new cell death process.

Pyroptosis differs from apoptosis and other types of cell death in morphology and mechanism. In contrast to apoptosis, the activation of pyroptosis is a pro-inflammatory reaction process^35^. Flow-cytometry analyses are frequently used to monitor the nature of cell death. These results from our present study through flow-cytometry analyses by using Annexin V-FITC/PI staining, showed that pyroptosis was triggered by *A. adenophora*. The discovery provided a rationale for explorring *A. adenophora* as an inducer of pyroptosis in mice hepatocyte.

In normal condition, inflammation is a defensive response of body to infection and tissue damage, which limits the damege to body^36^. Nevertheless, dysregulated and chronic inflammation may lead to secondary tissue damage by leukocytes, lymphocytes or collagen^37^. Inflammasome is a group of protein complexes composed of several proteins, including AIM2, NLRP3 and NLRC4. The assemble of inflammasome can be induced by diverse specific exogenous and endogenous factors^38^. Each inflammasome is activated by different signals factors. AIM2 inflammasome is activated merely by double-strand DNA, while NLRC4 is activated by specific bacterial proteins. Among the many known inflammasome complexes, the NLRP3 inflammasome is currently the best characterized one, which can be activated by exposure to a number of endogenous and exogenous irritants^39^. According to previous study, mitochondria-drived ROS was considered as a major sensor in NLRP3 formation^40^. NLRP3 inflammasome plays a decisive role in the process of activating caspase-1 and maturing IL-1β^41^. In the present study, the total level of ROS, core inflammation and immune factors including NLRP3, caspase-1 and IL-1β were all activated in a dose-dependent manner in liver from *A. adenophora*-treated mice. Moreover, the activity of caspase-1 was also elevated by *A. adenophora*, suggesting canonical pyroptosis pathway was activated through *A. adenophora* administration. Our present results demonstrated that NLPR3 may play a damage sensor in the process of hepatotoxicity induced by *A. adenophora*. Recent studies showed that GSDMD was a key executor in inflammatory caspase-induced pyroptosis^42–47^. On a single cleavage by caspase-1 at specific site in GSDMD, the N-terminal domain of GSDMD (GSDM-N) binds to membrane lipids and lyses cells through forming pores with an inner diameter about 10 nm on the membrane, which causes cell lysis and IL-1β release^19,44,45,48^. Notably, we found that the mRNA and protein levels of GSDMD were both increased by *A. adenophora*. GSDMD was cleaved into GSDM-N by different dose of *A. adenophora*, further demonstrate that pyroptosis was activated by *A. adenophora*. Moreover, NF-κB was also activated by *A. adenophora*. The NF-κB, as a cytosolic sensor, has a pivotal role in the activation of nuclear translocation and DNA binding. Previous researches have confirmed that NF-κB could combine with NLRP3 promoter, indicating that NF-κB has transcriptional regulation function on NLRP3^18,41^.

**Figure 6:**
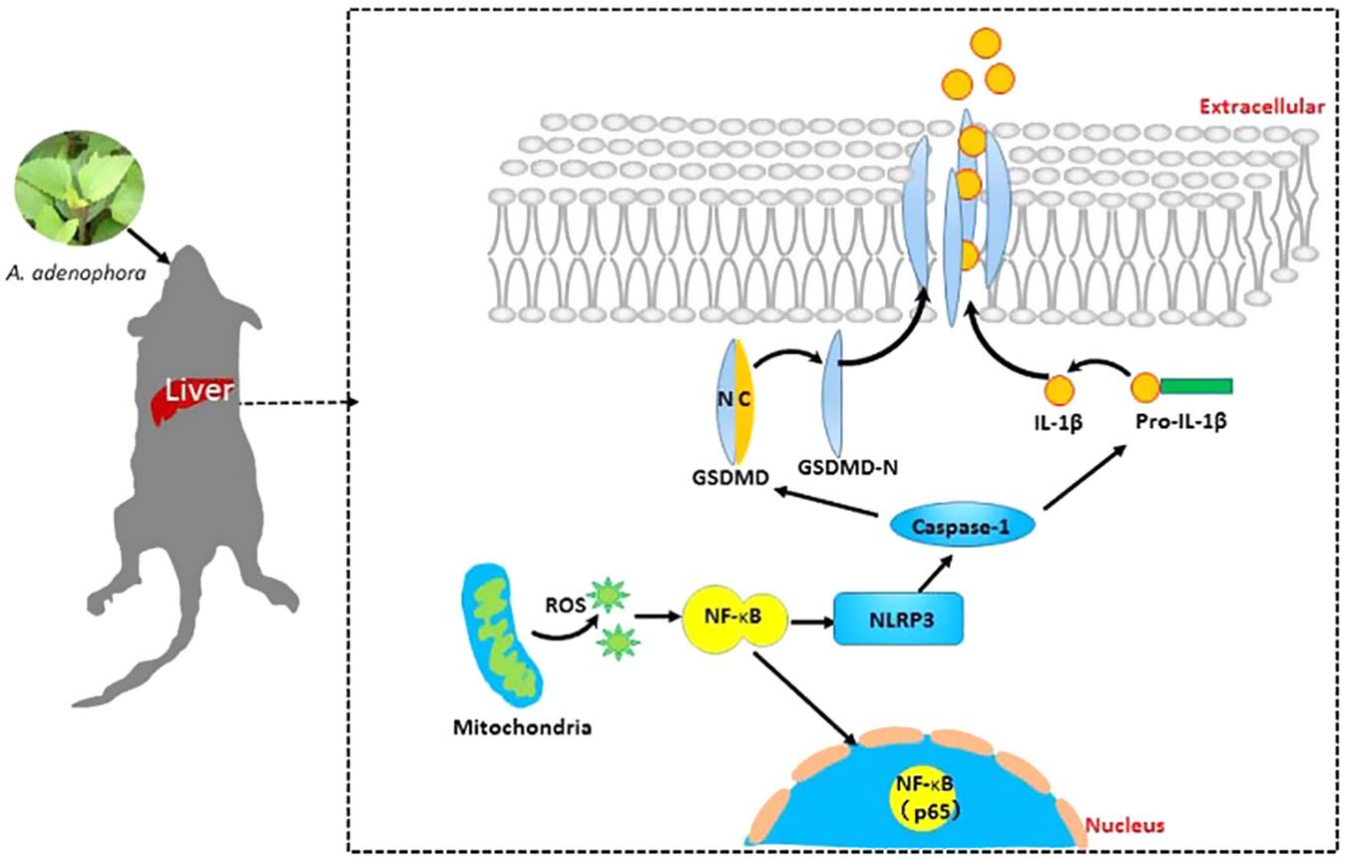
Schematic diagram of *A. adenophora*-induced hepatotoxicity and the underlying molecular mechanisms. *A. adenophora* promoted the generation of ROS in hepatocytes, then triggered NLRP3 and NF-κB activation, activated caspase-1-dependent pyroptosis pathway, resulting in liver inflammation and hepatotoxicity.

In conclusion, pyroptosis pathway was triggered by *A. adenophora* basing on ROS-NLRP3 activation, resulting in the hepatotoxicity in mice (Fig. 6). This study provided a novel insight into the hepatotoxicity caused by *A. adenophora* and the underlying mechanisms.

## Materials and Methods

### Ethics statement

This study was approved by Sichuan Agricultural University Animal Care and Use Committee (Approval No: 2012-024). All animal operation and procedures were conducted according to the approved guidelines and were in accordance with the international Guide for the Care and Use of Laboratory Animals.

### Animals experiment and plant materials

Eight-week-old Kunming mice were obtained from Chengdu Dashuo Experiment animal Co. Ltd. Forty female mice (average weight 19.70 ± 0.7 g) were divided into four groups (n = 10) at random, i.e. control group, group A, B and C. Mice in group A, B and C were fed with 10% (20 g/kg BW), 20% (40 g/kg BW) and 30% (60 g/kg BW) levels of *A. adenophora* pallet diet, respectively. Mice administrated with nutrient balanced feed were used as control (0%). Six weeks administration later, after weighting, all mice euthanized. The animals were housed in rooms with illuminated artificially with 12 h light/dark cycle, maintained ambient temperature at 22–24 °C as well as a relative humidity of 40–60%, respectively. Five mice were housed in per cage and administrated with diet at a dose of above-mentioned. Water was provided ad libitum. These measurements were repeated three times using different batches of mice.

*A. adenophoraA. adenophora* was collected from Dechang county, Sichuan Province, Southwest China in July 2017. The plants were dried in the shade and broken into power. And the ground power was stored in a dry environment before experiment.

### Biochemical analysis

The collected mice sera in different groups were used for biochemical test. ALT, AST and ALP kits were obtained from Mindray Medical International Ltd., China. And ALT, AST and ALP in serum were measured by BS-190 biochemistry analyzer (Mindray, Shenzhen, China) through different kinetic rate methods.

### ROS analysis

Cellular ROS level was measured with “Total ROS Assay Kit” (Thermo Fisher, USA) following the manufacturer’s protocol. Livers collected from mice were lightly ground and single cell suspensions were made through filtering a 300-mesh nylon screen. Hepatocytes was washed three times with pre-cold Phosphate buffer solution (PBS, pH 7.2–7.4) and then incubated with 1 × ROS assay stain solution at a concentration of 1 × 10^6^ cells/ml. Then hepatocytes were incubation at 37 °Cfor 20 min in the dark, and then were analyzed with FACS Calibur flow cytometer (Becton Dickinson, USA).

### Enzyme-linked Immunosorbent Assay (ELISA)

At the end of experiment, mice in each group were euthanized, and the sera were collected. Liver homogenized with PBS at a ratio of 1:9 (weight: volume) with a glass homogenizer in ice and centrifuged at 3,500 rpm at 4 °C for 10 min to obtain supernatant. IL-1β concentration in serum and liver supernatant from all experiment groups were detected by a commercial ELISA kit (Elabscience, Wuhan, China) according to the instructions strictly.

### Flow cytometry analysis

In order to determine the morphology of pyroptotic cells, the hepatocytes were made by the method above (See ROS analysis), and adjusted to concentration at 1 × 10^6^ cells/ml. Then, hepatocytes were stained by using Annexin V-FITC/PI Apoptosis Kits (Becton Dickinson, USA) following the manufacturer’s protocols. Then, stained cells were analysis with a FACS Calibur flow cytometer Becton Dickinson, USA) and the data acquisition and processing were carried out by Cell Quest software.

### Immunohistochemistry assay

After weighting, all mice were sacrificed humanely at the end of experiment. The livers in different groups were carefully dissected out, washed with cold PBS and fixed with 4% paraformaldehyde. Liver tissues were embedded by paraffin and were sliced into 5 μm sections. The sections were placed on slides coated with polylysine, then deparaffinized and rehydrated. The antigen retrieval was induced through heat Tris-EDTA buffer (pH 8). After cooling, section was blocked by 5% normal serum before incubation with the target primary antibody overnight at 4 °C. The next day, section was applied with biotinylated secondary antibody. Immunoreaction was observed by DAB as substrate.

### Activity detection of caspase-1

The Caspase-1 activities in all groups were performed by using colorimetric assay kits (Beyotime, Shanghai China) according to the manufacturer’s instruction. The assay kit is based on that acetyl-Tyr-Val-Asp *p*-nitroanilide (Ac-YVAD-*p*NA) can be catalyzed by caspase-1 to form *p*-nitroanilide (*p*NA), which has a strong absorption at 405 nm. The hepatocytes were collected and incubated in lysis buffer on ice for 30 min. And supernatant was collected after centrifugation, and protein concentration was measured by using “Modified Bradford Protein Assay Kit” (Sagon Biotech, Shanghai, China). Sample was incubated with substrate Ac-YVAD. The activity of caspase-1 was assessed by measuring the absorbance using a standard *p*NA curve.

### RNA extraction and quantitative real time PCR analysis

Total RNA from liver tissue was extracted with “Animal Total RNA Isolation Kit” (Sagon Biotech, Shanghai, China) by following the manufacturer’s procedure. Synthesis of single-stranded cDNA from RNA was performed according to the “PrimeScript™RT reagent Kit with gDNA Eraser” kit (TAKARA, Japan). Quantitative real time PCR (qRT-PCR) was performed using SYBR Premix ExTaq TM(TAKARA, Japan) by Thermal Cycler (CFX96, BIO RAD, USA). Relative gene expression was defined as a ratio of target gene expression versus β-actin gene expression. Gene expression values of control group in the experiment were used for gene expression calibration, respectively. The primers were synthesized by Sagon Biotech Ltd., (Shanghai, China), and the sequences were listed in Table S1. The β-actin, a house-keeping gene was introduced as an internal positive control standard for quantitative analysis. Data were analyzed with 2^−ΔΔ Ct^ method.

### Western blot analysis

Western blot was performed to detect the expression of target proteins. Total protein extraction was performed by using “Tissue or Cell Total Protein Extraction Kit” (Sagon Biotech, Shanghai, China). The liver tissues were lyzed in ice-cold buffer, and protein concentrations were measured by using “Modified Bradford Protein Assay Kit” (Sagon Biotech, Shanghai, China). Protein (40 μg) from each sample was separated by sodium dodecyl sulfate polyacrylamide gel electrophoresis (SDS-PAGE), and then transferred onto polyvinylidedne difluoride (PVDF) membrane. The membranes were blocked by 5% non-fat milk power which solved in 0.05% Tween 20/Tris-buffered saline (TBST) for 2 h at room temperature. Incubation with target primary antibodies was carried out overnight in 3% bovine serum albumin at 4 °C with gentle shaking. The next day, the membranes were incubated by appropriate secondary antibody for 1 h at room temperature. Immunoreactivity was visualized by chimiluminescence (Sagon Biotech, Shanghai, China). β-actin protein was introduced as a loading control.

### Statistical data analysis

All experiments were conducted with a minimum of three replicates, and the data were represented as means ± standards deviations. Statistical analysis was performed to compare *A. adenophora*-treated groups with the control group by using a one-way analysis of variance (ANOVA) complemented with the Turkey-Kramer multiple comparison test. And computations were performed by SPSS 22.0 software package (IBM, USA). All statistical artworks were performed using GraphPad Prizm 5.0. Statistical significance was considered at p < 0.05.

## Electronic supplementary material

Supplementary Data
