# Supplementary material for: Ageratina adenophora induces mice hepatotoxicity via ROS-NLRP3-mediated pyroptosis
Source: Sci Rep. 2018 Oct 30;8:16032. doi: 10.1038/s41598-018-34492-7 (PMC6207671; doi:10.1038/s41598-018-34492-7)
Supplement: Supplementary file 1 — Supplementary Data [file 41598_2018_34492_MOESM1_ESM.pdf]

Supplementary Data

Ageratina adenophora induce mice hepatotoxicity via ROS-NLRP3-mediated pyroptosis

Wei Sun [1,2,¶], Jie Fu [1,¶], Shanshan Liu [2,¶], Liwen Hu [1], Zhen Shi [1], Dong Yue [1], Zhihua Ren[1], Zhijun Zhong[1], Zhicai Zuo[1], Suizhong Cao[1], Guangneng Peng[1], Junliang Deng[1], and Yanchun Hu [1,*]

[1]Key Laboratory of Animal Disease and Human Health of Sichuan Province, College of Veterinary Medicine, Sichuan Agricultural University, Wenjiang District, Chengdu City, Sichuan, 611130, China.

[2]Tongren Polytechnic College, Bijiang District, Tongren City, Guizhou, 554300, China.

¶Wei Sun, Jie Fu and Shanshan Liu contributed equally to this work.

*Correspondence to Y.H. (email: hychun114@163.com).

Table S1. The primers sequences used for qRT-PCR

Items	Sense sequence	Antisense sequence	Product size
IL-1β	TCGCAGCAGCACATCAACAAGAG	TGCTCATGTCCTCATCCTGGAAGG	118
GSDMD	ACTGAGGTCCACAGCCAAGAGG	GCCACTCGGAATGCCAGGATG	140
Caspase-1	ACAACCACTCGTACACGTCTTGC	CCAGATCCTCCAGCAGCAACTTC	118
NF-κB	ATCCACCTCACCGGCCTCATC	TTCGCTGGCTAATGGCTTGCTC	162
NLRP3	GAGCTGGACCTCAGTGACAATGC	ACCAATGCGAGATCCTGACAACAC	146
β-actin	CCTAGGCACCAGGGTGTGAT	TCCATGTCGTCCCAGTTGGT	137

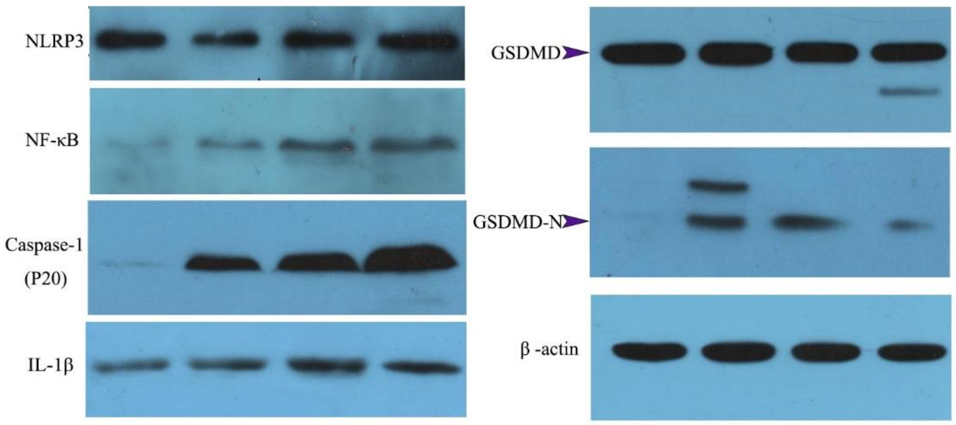

Figure-S1. Blots used for Figure 5a. All images were converteded into 8-bit type by using ImageJ software. The exposure time of all imgaes were range from 1 to 3 min. NLRP3 (114 kD), NF-κB (60 kD) and IL-1 β (17 kD) in panel I, II and VI were cropped from the same gel with exposure time of 2 mins. Caspase-1 p20 (45 kD), GSDMD (55 kD), GSDMD-N (35 kD) and β-actin (43 kD) were grouped in panel III, IV, V and VII, respectively.
